# Bots against corruption: Exploring the benefits and limitations of AI-based anti-corruption technology

**DOI:** 10.1007/s10611-023-10091-0

**Published:** 2023-03-25

**Authors:** Fernanda Odilla

**Affiliations:** Università Di Bologna, Bologna, Italy

**Keywords:** Accountability, Anti-Corruption, Artificial Intelligence, Brazil, Corruption, Integrity, Technology

## Abstract

Countries have been developing and deploying anti-corruption tools based on artificial intelligence with hopes of them having positive capabilities. Yet, we still lack empirical analyses of these automated systems designed to identify and curb corruption. Hence, this article explores novel data on 31 bottom-up and top-down initiatives in Brazil, presented as a case study. Methodologically, it uses a qualitative analysis and draws on secondary data and interviews to assess the most common features, usages and constraints of these tools. Data collected are scrutinised under a new conceptual framework that considers how these tools operate, who created them for what purpose, who uses and monitors these tools, what types of corruption they are targeting, and what their tangible outcomes are. Findings suggest that in Brazil, AI-based anti-corruption technology has been tailored by tech-savvy civil servants working for law enforcement agencies and by concerned citizens with tech skills to take over the key tasks of mining and crosschecking large datasets, aiming to monitor, identify, report and predict risks and flag suspicions related to clear-cut unlawful cases. The target is corruption in key governmental functions, mainly public spending. While most of the governmental tools still lack transparency, bottom-up initiatives struggle to expand their scope due to high dependence on and limited access to open data. Because this new technology is seen as supporting human action, a low level of concern related to biased codes has been observed.

## Introduction

Alice, Agata, Monica, Esmeralda, Iris, Rosie and Rui are, despite their names, non-humans. They were designed by humans to execute tasks previously reserved for humans; they can cross-check data faster and visualise and communicate them better, helping to identify or even predict anomalies related to different types of corruption in Brazil. They all are bots developed both bottom-up and top-down to work as anti-corruption tools to identify suspicious activities related to, for example, bid-rigging, fraud in contracts, cartel practices, the misuse of public money by congressional representatives, and sluggishness in the Supreme Court. These anti-corruption bots dig into data to increase accountability. By mapping these initiatives that have different levels of automation and functionalities, this paper aims to explore the benefits and limitations of using AI technology to fight corruption and improve integrity. In doing so, it questions what jobs these bots have been performing in the fight against corruption.

Like many tech buzzwords, bots and other applications of intelligent systems create great expectations for solving complex issues but attract a high level of distrust and generate debate. This is particularly the case with AI, still a vague concept that does not delineate specific technological advances and deals with subjective tasks classified as intelligent (Lanier & Weyl, 2020). Although no definition of AI has been agreed upon (Nilsson, 2009), the existing attempts have been criticised for being too anthropocentric since other forms of intelligence exist than human-specific (Koos, 2018; Wang, 2019). Yet AI is doubtlessly expected to extend the human and non-human limits of current performance in data processing and analysis, including being used as a powerful anti-corruption tool (Adam & Fazekas, 2021; Aarvik, 2019; Wirtz & Müller, 2019; Köbis et al., 2021, 2022).

Like the widely disputed concept of corruption, AI is an umbrella term. While corruption often has a hidden nature and encompasses a wide range of conducts and practices linked to the broad definition of misuse of entrusted power for private benefit (Johnston & Fritzen, 2020; Mungiu-Pippidi & Fazekas, 2020), AI includes, but is not limited to, different techniques, among them theorem proving, Bayesian networks, data mining, machine and deep learning, and functionalities such as representation, planning, reasoning, language, and image processing (Wang, 2019). As we can see, certain techniques overlap with tasks and their applications, adding complexity to attempts to capture all the features of AI. In the still incipient research of AI in the anti-corruption field, the ability to act autonomously, with or without human supervision, is what differentiates AI from ‘classic’ static and communication technologies, according to Köbis et al. (2021:3).

AI anti-corruption tools (henceforth, AI-ACT) are understood here as data processing systems driven by tasks or problems designed to, with a degree of autonomy, identify, predict, summarise, and/or communicate actions related to the misuse of position, information and/or resources aimed at private gain at the expense of the collective good. This type of application implies the analysis of a given environment based on a set of predefined rules before acting. It currently has the potential to work both autonomously or collaboratively with other machines and/or humans. This paper explores the most common features, affordances, and constraints of the initiatives of these emerging technologies that are already in use as anti-corruption tools.

So far, research on AI-ACT has attracted different fields and focused on the use of AI more in the private sector than in the public sector and by civil society, as noted by Neves et al. (2019). Overall, researchers have been discussing the potential of AI and other emerging digital technologies to fight corruption and/or improve accountability and transparency (Sturges, 2004; Bertot et al., 2010; Aarvik, 2019; Köbis et al., 2021, 2022) without forgetting that they can provide new corruption opportunities (Adam & Fazekas, 2021). While scholars have explored the use of AI-ACT, mainly in audit activities (Ghedini Ralha & Sarmento Silva, 2012; Neves et al., 2019; Taurion, 2016), Köbis et al. (2022) investigated existing challenges regarding data, algorithms and human–machine interactions and discussed the risks of using this type of technology in reinforcing existing power structures.

In addition, some studies apply AI techniques to predict and explain corruption across countries (Lima & Delen, 2020) or in public contracts (López-Iturriaga & Sanz, 2018), to detect fraud, corruption, and collusion in international development contracts (Grace et al., 2016), and to identify self-reported experiences with corruption on Twitter by using unsupervised machine learning (Li et al., 2020). In turn, the development and use of AI-ACT regarding bottom-up initiatives remain scarce. ProZorro in Ukraine (Aarvik, 2019) and Rosie, the Brazilian bot created by Operação Serenata de Amor (Köbis et al., 2022; Mattoni, 2020; Savaget et al., 2019), are among the few civil society initiatives that have received academic attention. Even fewer studies have looked at the outcomes of emerging technologies from the bottom up. Freire et al. (2020) are among the few who have not only questioned the impact of bottom-up monitoring on public service performance but also provided evidence of the null effect of a crowdsourcing mobile phone application and a Twitter bot (*Tá de Pé*^1^), both developed by the NGO Transparência Brasil, to allow oversight of school construction projects in Brazilian municipalities.

Aiming to contribute to this growing literature, this article provides an empirical analysis of bottom-up and top-down AI-ACT initiatives using Brazil as a case study. The theory combines the frameworks provided by Köbis et al. (2021) and Wirtz and Müller (2019) to introduce a conceptual framework tailored to explore relevant features of AI-ACT. Accordingly, the paper examines not only actors who have developed and used the technologies and the data inputs and outputs and the ensemble techniques applied, but also their functionalities, the types of corruption under analysis, who audits the technology, and the preconditions and facilitators of success. Methodologically, the article uses qualitative analysis and draws on secondary data and interviews to map the development and uses of these tools in Brazil. The findings suggest that civil servants and the public are more likely to manufacture tools based on their specific goals without outsourcing the process of development of AI-ACTs to target corruption in key governmental functions, mainly in public spending.

## The theorisation of AI-based anti-corruption technology

The visionary work of pioneers including Warren S. McCulloch and Walter Pitts, Alan Turing, and John McCarthy, who is credited with coining the term ‘artificial intelligence’ during the Dartmouth Workshop in 1956, have influenced researchers, practitioners and investors from different fields, including the humanities (Frankish et al., 2014). Anti-corruption studies, however, lag in the rapid advances in AI and its applications. Discussions about how this emerging technology is being used and whether it has been helping win the fight against corruption are still emergent. This is not to say that no anti-corruption technological applications exist, among them AI-based tools in development, nor has the field of anti-corruption studies failed to recognise that the advancement of digital technology offers more opportunities for promoting integrity, creating transparency, and fostering accountability. On the contrary, as mentioned earlier, there are both empirical evidence and theoretical assertions that emerging technologies, such as crowdsourcing and whistleblowing platforms, transparency portals, e-governmental services, social media, blockchain and AI, to name just a few, have the potential to work as effective anti-corruption and pro-accountability tools (see Shim & Eom, 2008; Bertot et al., 2010; Davies & Fumega, 2014; Mattoni, 2020; Adam & Fazekas, 2021; Köbis et al., 2022). Sanchez-Graells (2021) is one of the few voices to stress that AI-ACT, at least at its current narrow stage, can promote incremental improvements but not the expected significant transformation in the fight against corruption in public procurement.

Attention has been devoted to understanding under which contexts technological interventions, including AI, are more or less likely to work (Adam & Fazekas, 2021; Freire et al., 2020), which types of constraints and resistance they may encounter among users, such as auditors and inspectors (Neves et al., 2019), and when information and communication technology (ICT) tools have a high or low impact (Peixoto & Fox, 2016) or can be used to engage in corruption (Adam & Fazekas, 2021). However, different from more traditional ICTs, AI-ACT remains to be explored in depth, starting from its conceptualisation, main functionalities, and ethical considerations based on empirical evidence.

When more broadly reviewing the literature on AI models applied to public management, Wirtz and Müller (2019) presented an AI framework identifying three layers: AI technology infrastructure, AI functionality, and AI applications and services. To them, the cornerstone is the technology infrastructure because it determines how data is acquired, processed, and embedded into the greater system of AI-controlled applications (Wirtz & Müller, 2019:1078). Their AI functional layer considers the connection and cooperation of specific parts of hardware and software, and the applications and service layer deals with the interconnection and interrelation of AI techniques converted into toolboxes and/or devices. In turn, Köbis et al. (2021) offer an applied analysis of anti-corruption by outlining in detail how AI-ACTs present different potentials and pitfalls. They separate bottom-up and top-down approaches to highlight the potential of different actors, data inputs, algorithmic design, and institutional implementation involved in each of these approaches. Although the role of different actors and the importance of keeping humans in the loop are equally noted, developers and users are not separated, and little discussion appears concerning biases, transparency and auditing regarding the algorithms used against corruption in their work.

Thus, the framework presented next is an attempt to expand Köbis, Starke and Rahwan’s work. It focuses on the differences between the bottom-up and top-down approaches. The bottom-up and top-down approaches are defined by who developed the initiative and not by who is using it. The framework introduced below uses an adapted version of Wirtz and Müller’s layers to explore the human and non-human elements of the AI-ACTs. It is a framework developed to assess major elements of AI-ACTs in terms of their purposes, feasibility, performances, fairness and legitimacy.

**Fig. 1:**
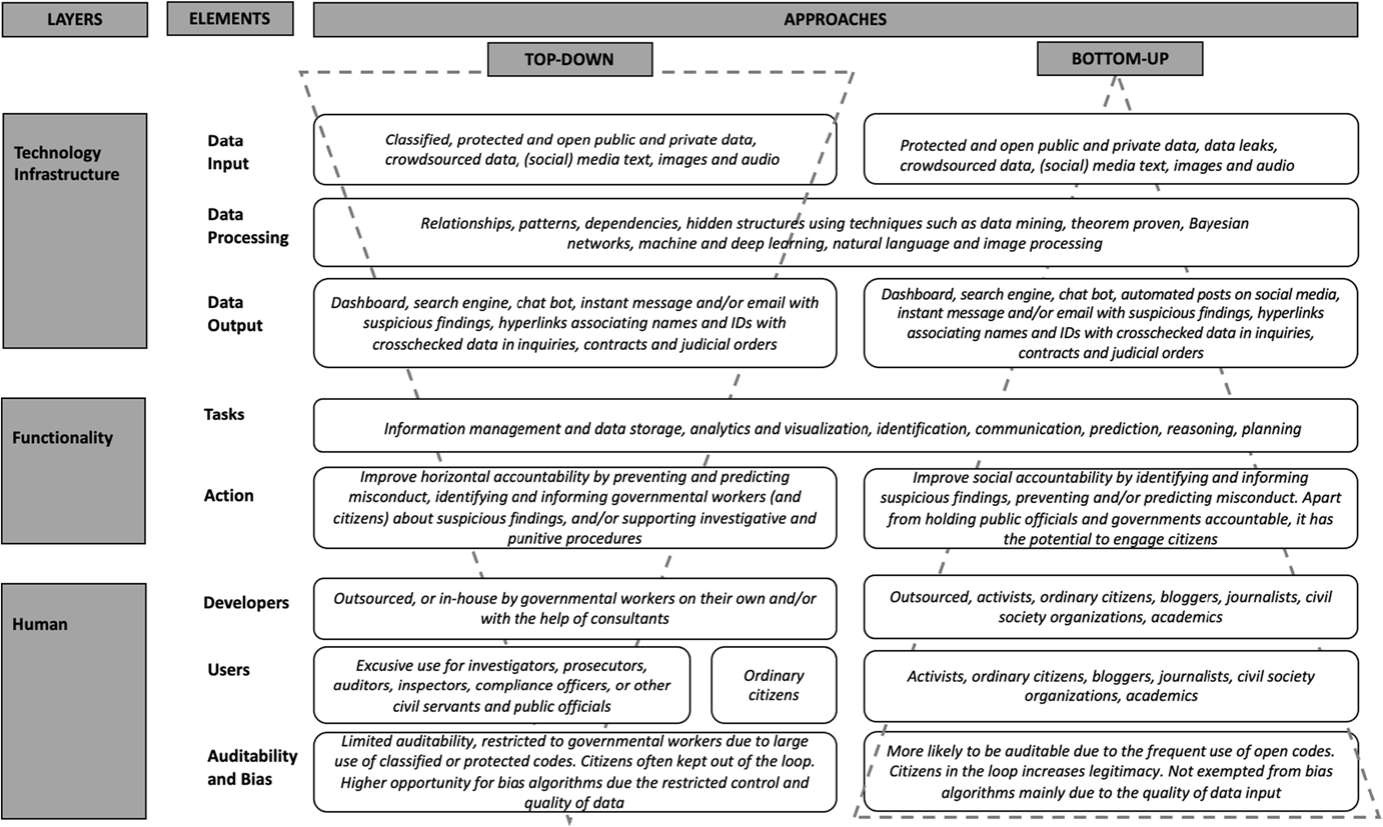
Conceptual framework for assessing artificial intelligence as an anti-corruption tool (AI-ACT) and its key components. Source: Author, based on and Wirtz and Müller (2019) and Köbis et al. (2021).

Figure 1 illustrates the layers and the elements in both bottom-up and top-down approaches, defined here as the key components of an AI-ACT, along with examples of their applications, to better understand what job these technologies have been performing in combating corruption.

As illustrated in Fig. 1, AI-based initiatives enjoy a high degree of diversity regarding technical acuteness, scope, and capacity, and AI is in a permanent state of development, testing and improvement. Based on the processes, functionalities and actors involved in AI-ACT, we can identify three layers: (1) technology infrastructure, (2) functionality, and (3) human, as detailed next. To the discussion of this conceptual framework, we add a fourth layer: key preconditions and facilitators of performance.

### The technology infrastructure layer

As noted by Wirtz and Müller (2019), technology infrastructure is the cornerstone of AI because it includes the input, processing and outputs of a wide range of data. The starting point is data acquisition, and this input is crucial for the quality of any expected outcome. The lack of digital, reliable, and consistent data, preferably in abundance and ready to be read by machines, will compromise the outputs. Processes can often be run with both high- and low-quality data, and can even identify patterns, relationships, dependencies, or hidden structures, the quality of which will be equally high or low. In addition, depending on how the data are processed, it is possible to identify in this layer the tasks that apply more or less advanced techniques and the subfields of AI, such as machine learning, natural language processing, and speech recognition and/or computer vision. Depending on the techniques applied and the data available, not only can the output vary, but also its accuracy and robustness. This is because, after data are acquired and processed, they are embedded into the greater system of applications that can return different outcomes in different digital formats. In the anti-corruption field, common outputs are updatable dashboards, risk-tracking and reporting instant messages indicating, for example, the probability of risky tenders or a suspicious expenditure; search engines for network analysis indicating the relationship between individuals and organisations, and voice assistants or bots able to chat to offer information or retrieve or reject reports exposing corruption-related cases.

### The functional layer

This layer encompasses and combines structures related to software and hardware able to execute their functions. However, it is more than a mere extension of the processes of data processing and embedding. The functional layer is concerned with the major purposes, i.e., the tasks it has been designed to execute, and/or the problems to be solved, and the efforts taken to achieve an aim. One could argue that it is a more subjective feature, but the functional layer works as an indicator of how autonomous a given initiative aims to be in terms of helping or replacing human action. The tasks can vary from information management and data storage to analytics and visualisation, to planning, communication, or even prediction, depending on the data available and the techniques applied. This is a different use of the concept compared to Wirtz and Müller (2019), who considered the functional layer as the moment when data are revised and analysed by a data processor connected to the inference engine able to draw conclusions and deduce potential courses of action. Although given tasks may overlap in bottom-up and top-down approaches, the functional layer allows AI-ACTs to act to improve both social and horizontal accountability mechanisms, i.e., mechanisms related to prevention, investigation and sanctioning coming from civil society or within governmental agencies.

### The human layer

The human layer covers key actors, their roles and their ethical concerns when developing, using and testing the fairness and accuracy of AI-ACT. While AI-ACT can be developed and used by ordinary people, activists, and journalists to hold public officials and governments to account from below, the top-down approach is often used to improve horizontal accountability and internal control within and between governmental agencies. Top-down efforts are more likely to include using tools to better communicate with civil society, obtaining information and complaints, and scrutinising governmental workers who abuse their position and citizens who attempt to engage in fraud, graft or other spurious transactions involving the government. In addition, it is crucial to have systems we can verify and trust. In this sense, humans need to pursue transparency and accountability for machines. As noted, algorithmic bias is often inherited from the datasets used as inputs. Therefore, the presence of open data and open codes allows not only the free use and redistribution of the datasets but also the implementation of collaborative improvements and auditing to know what decisions have been made or influenced by an AI. However, the governmental technological apparatus deals with confidential and protected data and with authorities claiming security concerns and often refusing to share, exchange, or disclose certain types of data. Under special circumstances, it may be necessary to have sealed terms of agreements to access certain types of infrastructures, and third-party auditors and/or civil society organisations can act on behalf of those individuals who do not have the skills or interest in holding machines accountable. The most important thing is having codes that are as explainable and as easily accessible as possible for humans.

### Key preconditions and facilitators of impact

Access to digital resources and technological skills are preconditions to developing and/or using AI-based tools, including ACTs, although reforms in the anti-corruption field seem to be submitted to stronger contextual constraints and limitations in terms of power relations and administrative capacities (Adam & Fazekas, 2021). In addition, as mentioned, corruption has a hidden nature, which, per se, creates issues to generate quality corruption databases. Corruption encompasses different types of conduct that are context-dependent and not always unlawful. Therefore, tools tend to be developed by design based on a corruption-related context and the quality, abundance, availability and accessibility of digital devices and specific datasets.^2^ To identify facilitators of AI-ACT impacts, it is necessary to go beyond the most common metrics used by developers, which are often linked to statistical measurements related to speed, probability and correct predictions (e.g., accuracy and sensitivity). Following Adam and Fazekas’ (2021) approach, we consider the impacts on corruption promoted via *administrative processes* by automating and standardising administrative tasks that may eliminate potential corrupt interactions or facilitate corruption detection, via *upward and downward transparency* to offer more access to information and improve accountability mechanisms, and via *collective actions* since digital media often lower costs for spreading information, creating awareness and fostering engagement in anti-corruption actions. We, however, avoid distinguishing grand and petty corruption, as the authors do, because this dualism often deals with blurred or overlapping situations related to both financial sums and individuals’ status or rank. Yet, the use of concepts like “success” should also be avoided as they can be seen as relative concepts that imply high levels of subjectivity that are hard to assess. Instead, impact is observed here through focal points linked to specific types of corruption, and from which tangible outcomes are achievable.

## Research design

The AI-ACT framework and its core components, as introduced in Fig. 1, will be applied to analyse the key characteristics of the bots against corruption operating in Brazil (see Appendix). The framework allows the assessment of the mentioned layers to evaluate which types of corruption each initiative targets and how autonomous the Brazilian bots are regarding human participation.

### Country context

Brazil is used as a case study for four reasons. First, although corruption scandals are present in all branches at the federal, state, and local levels, the country has been inching towards accountability (Power & Taylor, 2011). Second, the overall use of technology by both public administration and civil society has been growing rapidly in the country and is proving to be an important source of development (Malaquias et al., 2017). Third, the digital divide between individuals is rapidly decreasing among Brazilians, although we know digital illiteracy still poses challenges regarding access to technology (Nishijima et al., 2017). Finally, Brazil is a rich source of empirical data as it has been developing a considerable range of both bottom-up and top-down AI-based anti-corruption initiatives, especially at the national level (Carvalho, 2020; Dalben, 2020; Digiampietri et al., 2008; Ghedini Ralha & Sarmento Silva, 2012; Neves et al., 2019; Savaget et al., 2019).

In the mid-1980s, Brazil started adopting incremental anti-corruption reforms that resulted in institutional and legal advances, often driven by corruption scandals and domestic and international pressure. To fight corruption and maladministration, the country became more open and digital. As Da Ros and Taylor (2022: 55) note, a variety of laws have enhanced data accessibility in Brazil, including the 1991 Archives Law that determined the state as responsible for preserving and making available documents to the public, laws that required active transparency, the timely reporting of public spending and budget disclosures in open transparency portals, and the 2011 Access to Information Law allowing the public to request information and receive it in electronic and open formats if the request specifies it.

Other critical areas, such as campaign finance, public procurement, court procedures and administrative sanctions, have been regulated, and public information on these topics has become more transparent and available in organised digital databases. Even if these advances did not eliminate multiple issues related to data secrecy and accessibility, they set a high bar for transparency (Da Ros & Taylor, 2022), although the enactment of the General Data Protection Law (Law 13,709/2018) has been promoting data access reconsiderations. Additionally, in the past decade, governmental agencies have been investing in training and innovation, including launching public contests to promote best practices and tools, hackathons to design anti-corruption proofs of concept together with tech-savvy citizens, and setting up innovation labs to develop such proofs of concept, including AI-ACTs. However, Brazil still lags in its provisions on AI regulation and governance. The Brazilian AI Strategy, launched in 2021, seems more like a protocol of intent than an actual plan of action, and a recent survey showed that 72% of 263 governmental agencies consulted do not use AI-based tools (TCU, 2022).

### Data collection and analysis

Although this article does not provide an exhaustive list of initiatives, the 31 cases, 23 of which were developed by governmental agencies, one outsourced to IBM, and seven created by civil society, were selected based on functionalities that have digital anti-corruption features at their core. Thus, we did not list a series of AI-based tools, among them the Court of Accounts’ *Geocontrole*, which uses satellite images to recognise, for example, mines and dam-reservoirs, and to check if their activity is irregular, and the Revenue Service’s travellers control system, which includes facial recognition and a geoprocessing tool to identify a predefined list of passengers of interest associated with, for example, smuggling and drug trafficking to be selected for inspection (Jambreiro Filho, 2019).^3^

The selection criteria for cases were that they must: use any AI element according to the definition presented in the introduction, be operative in 2020, and have anti-corruption elements as their core functional layer. Cases that were still prototypes or were initiatives that had not been implemented in daily practice by 2021 were not included, such as the project MARA.^4^ Apart from the *Bem-Te-Vi* tool that selects analysts and justices to evaluate procedures at the Superior Labour Court based on their expertise and lack of declared conflict of interests regarding previous similar cases, we did not include any tools developed by the Brazilian judiciary, which is investing heavily in AI-based solutions to better manage court procedures but without the primary goal of curbing corruption (for a detailed list of the 64 AI-projects in 47 courts, see Salomão, 2020).

Tables 1 and 2 list the selected tools, their main sources of data and their goals, ordered by the year they were officially launched.

**Table 1:** Selected AI-based anti-corruption tool

AI-ACT			
*Name*	*Launch (year, by)*	*Main sources of data*	*Main goals*
*ContÁgil*	2009, Revenue Service	Sensitive and protected data on asset declarations, sets of books and invoices, ownership registers and tax payments	Data clustering, outlier detection, topic discovery, and co-reference resolution features for retrieving and analysing anomalies and building network graphs with people, companies and their relationships to support financial audit activities
*Aniita (Analisador Inteligente e Integrado de Transações Aduaneiras, Intelligent and Integrated Analyser of Customs Transactions) and BatDoc (document mismatch detector)*	2012, Federal Revenue Service (Receita Federal)	Sensitive and protected data such as import declarations and digital images of auxiliary documents (e.g., invoices and bills of lading)	Identify anomalies related to cases of potential tax evasion and tax avoidance in customs. BatDoc is a tab of Aniita that applies optical character recognition to digital images to extract information from auxiliary documents
*Sisam (Sistema de Seleção Aduaneira por Aprendizado de Máquina, customs selection system through machine learning)*	2014, Federal Revenue Service (Receita Federal)	Sensitive and highly protected economic, financial, tax and customs datasets	Learn both from inspected and non-inspected import declarations, calculate the probability of about 30 types of errors for each declaration, generate a dashboard and write a report based on natural language presenting the findings to support customs inspectors' work
*Projeto Cérebro (Brain Project)*	2015, Council for Economic Defense (CADE)	Public databases related to ownership registers and public procurements	Generate a dashboard signalling any potential risk and/or sign of violation of competition law
*Alice (Análise de Licitações e Editais, Analysis of Biddings and Call for Bids)*	2015, Office of the Comptroller General (CGU) and later improved by the Federal Court of Accounts (TCU)	Tenders published on Comprasnet (public procurement portal), Official Gazette of the Federal Republic of Brazil, sanctions and ownership databases	Promote daily search for key information to identify potential irregularities, generate risk alerts by email and data visualization on the dashboard before public bidding awards or signing of contracts
*Rosie and Jarbas*	2016, Operação Serenata de Amor (Love Serenade Operation)-Open Knowledge Brasil	Public data on congressional spending and attendance, ownership registers, and private data on Google, Foursquare, and Yelp	Estimate a “probability of corruption” based on standard deviations for each reimbursement receipt submitted by MPs; generate a dashboard and tweet suspicious findings, asking followers to check them
RobOps	2016, Instituto OPS	Open and public data on the spending of congresspeople and the fiscal condition of companies at the Revenue Service	Improve transparency and promote social accountability by displaying the expenditures ranking the politicians on a dashboard and tweeting every 15 min
*Iris (Indicator de Risco de Irregularidades, or Irregularities Risk Indicator)*	2017, Court of Accounts of the State of Rio de Janeiro (TCE-RJ)	Sensitive, protected, and open data related to public procurement and data on business ownership and aerial and street-level (Google) images, provided by municipalities and the State of Rio de Janeiro	Establish relationship networks and calculate the probability of risk factors visualized on a dashboard
*Monica (Monitoramento Integrado para o Controle de Aquisições, or Integrated Monitoring for Procurement Control)*	2017, Federal Court of Accounts (TCU)	Public procurement data in the federal legislative, judicial and executive branches and the prosecution service	Offer data visualization tools with filters and the possibility to download spreadsheets with information to monitor public procurement in the federal judicial and legislative branches
*Watson*	2017, IBM	All databases available at the federal police in the State of Rio Grande do Sul	Index texts, allow searches regarding investigative procedures based on data mining, and offer a dashboard with questions and answers to support police officers' work
*Ajna (Plataforma de Visão Computacional e Aprendizado de Máquina, Computer Vision and Machine Learning Platform)*	2017, Revenue Service	X-ray images of all containers leaving or entering the country at the port of Santos, with corresponding declarations presented to the customs clearance department	Identify patterns, classify and predict risks and warn of anomalies to control fraud by using computer vision, data mining and optical character recognition techniques to identify possible irregularities in cargo containers and transhipments
*Ta de Pé*	2017, Transparência Brasil	Text message of users, public official database of school construction sites including geolocation, timeline and money transfers, and automatic alerts to governmental bodies and their respective replies	Allow citizens to monitor school constructions in Brazilian municipalities initially via a mobile phone app that was later converted into a WhatsApp chatbot. Promote alerts and authorities’ replies via a bot on Twitter
*Adele (Análise de Disputa em Licitações Eletrônica, Dispute Analysis in Electronic Bidding)*	2018, Federal Court of Accounts (TCU)	Protected and sensitive data on electronic bidding, such as the IPs of the bid participants and data on companies and individuals	Inform inconsistencies and anomalies in electronic public bidding in the form of a dynamic dashboard
*Zello (chatbot)*	2018, Federal Court of Accounts (TCU)	Users’ text messages, public auditing procedures, names of those sanctioned and acquitted, and Q&A list on the functioning of the TCU	Chat via text messages and guide citizens to access information on the TCU, including auditing procedures and clearance certificates of any individual
*Detecção de Irregularidades em Benefícios (Detecting Irregularities in Benefits, DIB)*	2018, Governmental Social Security Technology and Information Company (DataPrev)	Governmental datasets, including protected social, labour, and pension governmental data	Identify patterns and signal risks of social benefit programme fraud and suspicious payments by crossing large numbers of datasets and offering data visualization applications such as panels and storyboards
*Rui Barbot*	2018, JOTA news website	Metadata of procedures on the Supreme Court website	Control judicial delay by automatically signalling on Twitter and by email when court procedures are reaching 180 or 270 days or years without movement

**Table 2:** Selected AI-based anti-corruption tools

AI-ACT			
*Name*	*Launch (year, by)*	*Main sources of data*	*Main goals*
*Malha Fina de Convenios (Singling out agreements for inspections)*	2018, Office of the Comptroller General (CGU)	Public and protective data available on the distribution of federal funds and datasets of alerts of possible irregularities	Provide scores to measure the likelihood that accounts of beneficiaries of financial covenants can be rejected or not approved at the moment they are presented for examination and settlement
*Cida (Chatbot Interativo de Atendimento Cidadão, or Interactive Chatbot for Citizen Service)*	2018, Office of the Comptroller General (CGU)	Facebook and Telegram libraries, users’ text messages, and content available on Fala.BR (ombudsman) platform	Chat via text messages on social media and guide citizens who want to enter complaints, denunciations, or greetings or give feedback
*Esmeralda*	2019, Court of Accounts of the Municipalities of the Goiás State (TCM-GO)	Data related to public procurement, taxes and benefits received through a system created to transfer data from and to the municipalities in the State of Goiás, audited by the local Court of Accounts	Identify anomalies visualized on a dashboard and, via text messages, notify risks related to the purchase and payment of goods, services, workers, and benefits
*FARO ( Ferramenta de Análise de Risco em Ouvidoria, or Risk Analysis Tool for Ombudsman)*	2019, Office of the Comptroller General (CGU)	Complaints and denunciations received via the Fala.BR online system (governmental crowdsourcing platform)	Evaluate and classify complaints and denunciations as suitable, i.e., meeting the minimum requirements to justify the opening of an investigative procedure
*Ta de Pé Merenda*	2020, Transparência Brasil and Federal University of Campina Grande	Open public data from two State Courts of Accounts (Rio Grande do Sul and Pernambuco) and the Federal Revenue on its beta version	Monitor local purchases of food for schools via a website with a search engine and a chatbot for food quality control by students and school staff
*Ta de Pé Compras Emergenciais*	2020, Transparência Brasil	Open public data from two State Courts of Accounts (Rio Grande do Sul and Pernambuco) and the Federal Revenue on its beta version	Monitor emergency purchases related to COVID-19 via a website with a search engine
*Bem-te-vi*	2020, Superior Labour Court (TST)	Historical data of the justices' decisions from the last two years; labour legal language model built from data from 27 regional labour courts	Screen and forward labour cases for appreciation based on predictive models that consider the expertise and lack of conflict of interest of judiciary analysts and ministers
Publique-se	2020, Abraji and Transparency International Brazil	Open and public legal proceedings, including charges, reports, receipts and other documents that cite politicians as defendants or plaintiffs in the higher, federal and local courts	Monitor court procedures, increase transparency and facilitate access to official documents related to active and passive corruption and administrative improbity involving politicians, via a website with a search engine
*Agata (Aplicação Geradora de Análise Textual com Aprendizado, or App for Generating Textual Analysis with Learning)*	2020, Federal Court of Accounts (TCU)	Comprasnet (governmental procurement portal) and internal datasets	Refine the list of words of public procurement documents and their respective text contexts that may require further inspection by using humans to train the algorithm and incorporate them into other monitoring tools
*Sofia (Guidance System on Facts and Evidence for the Auditor)*	2020, Federal Court of Accounts (TCU)	Internal databases with individuals and business information, such as registers on ownership, death, sanctions and investigations	Review draft texts by verifying sources of reference, identifying correlations between the information written in the text and other procedures, and informing findings in comment boxes to support civil servants writing auditing reports
*Carina (Crawler e Analisador de Registros da Imprensa Nacional)*	2020, Federal Court of Accounts (TCU)	Official Gazette of the Federal Republic of Brazil	Identify anomalies related to urgent public health-related contracts and bids and offer a dashboard and email alerts with information about suspicious purchases
*Delphos*	2020, Federal Police	Public databases and sensitive databases with protected individual-level information such as names of people, companies, addresses, values of contracts and payments, emails, and telephone numbers	Make predictions and signal risks of crimes (including corruption) to support the work of the federal police
*﻿CoviData*	2020, TCU	Public and sensitive databases with individual-level information, including public bids, public contracts and previous sanctions, and a database with 200 pieces of news extracted from Google news and related to public procurement and names mentioned in these news items, such as companies, public institutions, names of people and places	Make predictions and provide risk scores for suppliers that participated in Covid-19-related public procurement processes to support the work of the Court of Accounts
*PLACC (Plataforma de Análises Cognitivas para o Controle, or Cognitive Analytics Platform for Control)*	Year not available, TCU	Criminal court procedures, the Revenue Service's ownership and taxes information, and other sensitive databases	Identify individuals and companies, extract relevant information about them, and establish relationship networks and money flows displayed in the form of graphs to support investigations and audits at the Court of Accounts
*DadosJusBR*	2020, Federal University of Campina Grande and Transparência Brasil	Open public data on gross earnings, bonuses and top-up salary payments in the bodies that are part of the Brazilian justice system	Increase transparency of salaries and benefits paid to judges and prosecutors and provide accessible data visualizations via a dashboard

Two types of data sets were used to select the top-down initiatives: 1) open public data related to the development and use of anti-corruption AI-based technology retrieved from a catalogue^5^ on algorithmic transparency in the Brazilian federal executive launched in 2021 by the NGO Transparência Brasil (2021a, 2021b), and 2) searchable databases of requests and answers about the use of AI made through the Access to Information Law (LAI) in two platforms, one run by the federal government and the other by the NGO.^6^ The bottom-up initiatives were mapped within the scope of the BIT-ACT (Bottom-Up Initiatives and Anti-Corruption Technologies) project conducted at the University of Bologna, based on desk research and expert interviews.

After the selection of initiatives, extra basic information was complemented through desk research. Bottom-up initiatives were collected on platforms such as GitHub, where open-source projects are often made available and are open for collaboration, and on messaging apps such as Telegram and Discord, where collaborators interact and exchange technical information. Documents published online and official presentations delivered by the developers, detailing the technology used by governmental and civil society initiatives that are available online, were used to analyse the cases of bottom-up and top-down initiatives. Other relevant or missing data regarding inputs, data process, outputs and accountability of top-down initiatives were collected through the Access to Information Law via formal requests submitted to each department responsible for the initiatives.

The initial response to a request for information submitted to the Federal Police mentioning one initiative (Watson) and requiring data regarding any other AI-ACT stated that no contract was in place regarding the use of AI or ML against corruption. After an appeal was filed, along with links to three initiatives, based on videos and reports available online in which the use of AI tools was detailed by the Federal Police, partial access was granted, with incomplete answers regarding the three initiatives listed in the request. The Federal Police argued that such information could compromise their investigative and operational capacity and did not supply, for example, details of a tool named Delphos that had been developed by the forensics team to use language processing to cross-check data on people and companies to make diagnoses and suggest actions.^7^ The Court of Accounts of the State of Rio de Janeiro (TCE-RJ) did not grant even general information on data inputs and outputs, nor the software used for its tool named Iris, as it was classified as sensitive information. The fact that different agencies responded to the full questionnaire submitted via the ACT while others did not suggests a lack of standards, processes, and regulatory frameworks regarding the transparency of this type of information in public bodies.

The data collected were then complemented by 13 in-depth interviews with experts (2), developers of these types of technology who are governmental workers (4), and ordinary tech-savvy citizens (7) involved in the case of grassroots initiatives. One written and 12 online interviews were conducted in Portuguese between June 2020 and March 2021. The interviewees were pseudonymised. All the interviewees were technical developers and they were asked about how they became interested in digital anti-corruption tools and their views on the current uses, limitations and challenges of these technologies. Those who created AI-ACTs were asked to explain their tools in terms of inputs, processing, outputs, and the main functionalities and to detail the process of their development. In addition, topics such as potential bias and fairness of AI were approached. MAXQDA 2020 was used for the analysis of the interviews, which employed a coding scheme that followed a deductive strategy and drew on the analytical framework outlined in the previous section, which guided the data collection and analysis of all the materials used in this exploratory research. However, since the interviews were conducted to complement the data already collected on the AI-ACTs, their analysis was limited to identifying certain traits and topics repeated by the respondents.

## Findings and discussion

Since the Revenue Service first launched its pioneering AI-based tools ContÁgil and Aniita, in 2009 and 2012, respectively, to automate and standardise administrative tasks conducted by its inspectors, other AI-ACT initiatives have emerged. Brazil has created a web of accountability between governmental agencies whose attributes compete with and complement each other regarding oversight, and investigative and sanctioning tasks (Power & Taylor, 2011). The governmental agencies investing more in AI-ACT as part of this web of accountability include the already mentioned Revenue Service, the Federal Court of Accounts (*Tribunal de Contas da União*), the Office of the Comptroller General (*Controladoria Geral da União*, CGU), and the Administrative Council for Economic Defence (CADE). The Federal Police, as mentioned before, are less open about how they use AI-ACT, although we know they have been using it. The judiciary has been investing in AI-based tools to speed up administrative proceedings.

Most of the tools analysed here target corruption in key governmental functions, such as public expenditure, public revenue, and court procedures and practices, as Tables 3, 4 and 5 below illustrate. AI-ACTs developed by law enforcement agencies are limited to specific areas that are part of their main tasks and responsibilities. For example, the Revenue Service is responsible for the intake of the government’s revenue, including the customs department, and, therefore, three of its tools mapped by this study focus on curbing fraud in clearance procedures. Courts of accounts are responsible for auditing the accounts and overseeing the implementation of budgets, and these agencies have been investing in AI to help their inspectors check cases of non-compliance with public procurement rules and mismanagement of public funds. The same applies to the CADE and the Social Benefit Service (INSS), whose innovative technologies fit with their attributions, i.e., curbing cartel practices and benefit frauds, respectively. Public spending is the focal point of most of the bottom-up initiatives analysed, although their tools cover areas outside the scope of governmental initiatives such as the construction of schools and the purchase of food for schools, and congressional representatives’ expenditures, as detailed in Table 3,

**Table 3:** Mapping the main features of Brazilian AI-ACTs used to monitor public expenditures

Specific areas	Focal point (Number of initiatives)	Name of the AI-ACT/Developer	Approach	Users	Promote impact via	Main weaknesses observed
*Public expenditures*	Misspending of congressional representatives (2)	Rosie and Jarbas/Operação Serenata de Amor; RobOps/Operação Política Supervisionada	Bottom-up	Any citizen with access to the internet and/or a Twitter account	Promoting upward transparency and collective actions	Limited open data to expand the scope; issues to engage ordinary citizens and to maintain the tool on a volunteered basis
	Issues with public tenders and contracts for school meals (1)	Tá de Pé Merenda/Transparência Brasil and Federal University of Campina Grande	Bottom-up	Any citizen with access to the internet	Promoting upward transparency and collective actions	Limited open data; tool restricted to two states; issues to engage ordinary citizens
	Pandemic emergency purchases irregularities (2)	Tá de Pé Compras Emergenciais/Transparência Brasil	Bottom-up	Any citizen with access to the internet	Promoting upward transparency and collective actions	Limited open data to expand the scope; issues to engage ordinary citizens; lack of use
		CovidData/TCU	Top-down	TCU auditors	Automatizing and standardizing administrative tasks	Lack of clear criteria to select the data (mainly items from Google news) and to score the risk; limited auditability
	Public school construction delays (1)	Tá de Pé/Transparência Brasil	Bottom-up	Any citizen with access to the internet, mobile and/or Twitter	Promoting upward transparency and collective actions	Limited open data to expand the scope; issues to engage ordinary citizens; lack of use
	Non-compliance with public procurement rules (8)	Alice, Carina, Monica, Agata, Adele, Sofia/TCU; Iris/TCE-RJ, Esmeralda/TCM-GO	Top-down	Auditors and workers of the respective agency that developed the tool	Automatizing and standardizing administrative tasks	Alert overflow and eventually lack of use due to a high number of false positives; slow adaptability to new types of wrongdoings; limited auditability; high risk of bias and unfairness
	Cartel practices (1)	Projeto Cérebro/CADE	Top-down	Auditors and workers of the respective agency that developed the tool	Automatizing and standardizing administrative tasks	Alert overflow and eventually lack of use due to a high number of false positives; slow adaptability to new types of wrongdoings; limited auditability; high risk of bias and unfairness
	Benefit fraud (1)	DIB/DataPrev and INSS	Top-down	INNS Inspectors and Auditors	Automatizing and standardizing administrative tasks	Alert overflow and eventually lack of use due to a high number of false positives; slow adaptability to new types of wrongdoings; limited auditability; high risk of bias and unfairness
	Mishandling of transferred federal funds (1)	Malha Filha de Convênios/CGU and Ministry of Economy	Top-down	Auditors from the CGU	Automatizing and standardizing administrative tasks	Alert overflow and eventually lack of use due to a high number of false positives; slow adaptability to new types of wrongdoings; limited auditability; high risk of bias and unfairness

**Table 4:** Mapping the main features of Brazilian AI-ACTs used to monitor governmental revenue, the management of public funds, and citizens’ complaints and denunciations

Specific areas	Focal point (Number of initiatives)	Name of the AI-ACT/Developer	Approach	Users	Promote impact via	Main weaknesses observed
*Public revenue (e.g., tax evasion or avoidance)*	Customs clearance issues (3)	Sisam, Aniita, Ajna/Revenue Service	Top-down	Revenue Service Inspectors	Automatizing and standardizing administrative tasks; assisting financial investigations	Not fully trusted by users; alert overflow; existing false positives; slow adaptability to new types of wrongdoing; limited auditability; high risk of bias and unfairness
	Incompatible financial transactions (1)	ContÁgil/Revenue Service	Top-down	Revenue Service Inspectors	Automatizing and standardizing administrative tasks; assisting financial investigations	Not fully trusted by users; alert overflow; existing false positives; slow adaptability to new types of wrongdoing; limited auditability; high risk of bias and unfairness
*Management of public funds and reports on finances*	Fiscal irresponsibility spotted and sanctioned by the Court of Accounts (1)	Zello Chatbot/TCU	Top-Down	Any citizen with access to Twitter and/or WhatsApp	Promoting downward transparency; automatizing and standardizing administrative tasks	Limited data; limited auditability; issues to engage the ordinary citizen; lack of use
*General*	Complaints and denunciations of any type of irregularity (2)	FARO/CGU	Top-down	Auditors from the CGU	Automatizing and standardizing administrative tasks; promoting upward transparency	Lack of use due to the high number of false positives; slow adaptability to new types of wrongdoing; limited auditability; risk of unfairness
		Cida Chatbot/CGU	Top-down	Any citizen with access to Facebook and Telegram	Automatizing and standardizing administrative tasks; promoting upward transparency	Limited data; issues to engage the ordinary citizen; lack of use

**Table 5:** Mapping the main features of Brazilian AI-ACTs to monitor criminal and court proceedings

Specific areas	Focal point (Number of initiatives)	Name of the AI-ACT/Developer	Approach	Users	Promote impact via	Main weaknesses observed
*Criminal procedures*	Charges of passive and active corruption against politicians (1)	Publique-se/Abraji, Transparency International Brazil and Digesto	Bottom-up	Any citizen with access to the internet	Promoting upward transparency and collective actions	Limited data (only higher courts and politicians who ran for office); risk of offering outdated information
	Investigation of corruption and correlated crimes and illicit activities (2)	Delphos/Federal Police and Watson/IBM	Top-down	Federal police officers and federal police officers in the State of Rio Grande do Sul	Automatizing and standardizing administrative tasks; Assisting criminal investigations	Limited auditability; high risk of bias and unfairness due to the quality of the data
	Depicts relations among individuals and companies mentioned in court cases (1)	PLACC/TCU	Top-down	Auditors from the TCU	Automatizing and standardizing administrative tasks; Assisting account procedures	Lack of clear criteria to select data; limited auditability; issues with ORC of digitalized documents
*Court proceedings and practices*	Inefficiency, delays and conflicts of interest in the Superior Labour Court (1)	Bem-Te-Vi/TST	Top-down	Civil servants from the Superior Labour Court	Automatizing and standardizing administrative tasks	Alert overflow and eventual lack of use; limited auditability; high risk of bias and unfairness
	Delays and inefficiency in the Supreme Court (1)	Rui Barbot/JOTA info news	Bottom-up	Any citizen with access to the internet and/or a Twitter account and JOTA journalists	Promoting upward transparency and collective actions	Limited data; lack of clear criteria to select data; issues to engage ordinary citizens; lack of use
	Salaries and benefits paid to judges and prosecutors (1)	DadosJusBR/ Federal University of Campina Grande and Transparência Brasil	Bottom-up	Any citizen with access to the internet	Promoting upward transparency and collective actions	Limited data; issues to engage ordinary citizens, lack of use

While most tools developed by civil society aim to impact corruption by improving upward accountability and transparency and promoting collective actions by creating awareness, most governmental tools automate and standardise administrative tasks under their prerogative. Governmental tools, therefore, are more likely to be designed to enhance efficiency, productivity and performance, and to support decision-making in risk identification and monitoring. Bottom-up initiatives aim to engage the public and interact with them; hence, they often publish their findings on social media, while governmental tools are more likely to be for government use. Three governmental tools, however, are improving downward transparency; they use automated systems to assist the public, a task that would often be performed by humans (see Table 4). In two of these cases, AI has been used by governmental agencies to improve government-public interaction via chatbots on social media: TCU’s Zello (on Twitter and Telegram) and CGU’s Cida (initially on Facebook and later Telegram). While Zello offers automated access to procedures and clearance certificates when names are provided, Cida offers to help the user report a denunciation, complaint, request, suggestion, greeting or even a request to simplify a public service to the ombudsmen at the federal executive. The third governmental tool designed to promote downward transparency and improve accountability is FARO, which deploys AI to evaluate elements of the public’s denunciations and complaints, decide whether to open investigative procedures and keep complainants informed about decisions taken.

Cida is a chatbot that engages and interacts with the public and is a crowdsourcing tool once it collects data provided by them. Curiously, the CGU does not consider Cida an AI-based tool, as they made explicit in their response to the request to access information for this study. *Tá de Pé*, equally not considered AI by its creators, was designed to crowdsource data via a chatbot, but only information related to the delayed construction of schools and nurseries may, in the end, be related to misuse or mismanagement of public money. This calls attention to the fact that Brazil has few crowdsourcing tools or digital channels dedicated to receiving accusations of corruption. However, tools dedicated to identifying and monitoring criminal investigations and court procedures exist, among them those related to unlawful conduct intended to secure benefits for oneself or another. Overall, all the tools have key weaknesses related to limited access to databases and lack of use, which is more salient in bottom-up AI-ACTs. Governmental tools offer limited auditability, which may increase the risk of bias and unfairness.

Most unusually, in-house solutions predominate, with both the public and civil servants developing their tech tools using the knowledge and skills of those with IT backgrounds. Among the sample analysed, only one tool, Watson from the Federal Police, was outsourced to IBM. Often, cases were developed by a small group of people or by just one person. In governmental initiatives, civil servants are encouraged to develop and maintain the AI-ACTs mainly by participating in tech meetings and innovation challenges, with taxpayers ultimately financing them. Bottom-up tools have been created by activists, concerned citizens or media outlets. Sources of funding, therefore, vary from crowdfunding, public and private grants, subscriptions, and/or ads, in the case of media outlets. The following subsections shed light on specific tools following layer-by-layer analysis according to the proposed theoretical framework.

### Data inputs, processing and outputs

Datasets used as inputs vary, but often bottom-up tools rely on open data and top-down on both open and protected data. Portals with information on e-procurement (e.g., ComprasNet), business ownership and previous sanctions have been used as main inputs by governmental initiatives. Some initiatives combine open public and private data. The bot Rosie, for example, processes public open databases made available by the Lower Chamber and Revenue Services and private open databases on Google, Foursquare, and Yelp to calculate distances and average prices. Interviewees highlighted how Brazil is advanced in active transparency regarding public data and has well-established legislation for access to information, especially in the federal sphere. They complained, though, that not all types of data are accessible or available, not even by law enforcement agencies, that not all systems are yet able to communicate among themselves, and that the level of partnership among public agencies is still low. Even the codes are not easily accessed, apart from code exchanges between the Federal Court of Accounts and the CGU. One great exception is the bot Alice, created in 2015 by CGU to help auditors curb corruption in public procurement, embezzlement, graft, and anti-competitive practices by analysing bid submissions, contracts and calls. One year after its inception, an agreement was signed to share the code with the TCU, which improved it by adding ML techniques (a random forest classifier) to the existing regular expressions (regex) to mine data daily from the Federal Official Gazette and the ComprasNet portal (Interviewee 8).

Considerable variation in data processing among the existing tools was observed, indicating a wide range of architectures that vary from web scraping to descriptive statistics to a more complex analysis that mixes different types of processing features. For example, Iris, from the Court of Accounts of the State of Rio de Janeiro (TCE-RJ), applies a multicriteria model based on an analytical hierarchy process to establish relationships and dependencies. It uses data mining, deep learning, and image processing to calculate risk factors for the auditors at the TCE. Among the processes in place, Iris uses Google services to transform addresses into geographic codes to generate aerial and street-level images of the companies participating in bidding processes. Based on these images, a model applying a convolutional neural network (CNN, or ConvNet) evaluates the probabilities of each being a ghost company, using the Google library and model (Tensor Flow and Inception v3, respectively) for deep learning. Although it was developed in-house, it deployed ‘ready-to-use’ tutorials, scripts and libraries.

To identify cases of benefit fraud, Dataprev developed a tool based on data mining and statistical analyses with protected governmental datasets using Python and SAS Miner. It uses techniques such as deep learning by applying CNN and a sample of 3,000 to 6,000 benefits with which to train the algorithm. The Revenue Service’s Sisam, in turn, learns both from inspected and non-inspected import declarations to calculate approximately 30 types of potential errors in each import declaration (Interviewee 1). It is a set of Bayesian networks programmed in Java whose conditional probability tables have been replaced by smoothing hierarchies to identify possible false descriptions of goods, missing licences, and wrong preferential tax claims that help inspectors decide which imports and exports to check.

For data processing, however, not all initiatives use ML or vision recognition. Some use less sophisticated techniques, such as automated data mining and crosschecking data using descriptive statistics and filters to highlight risks and anomalies. *Projeto Cérebro* (the Brain Project) and the CADE, created in 2015, do this to help flag cases of anti-competitive practices, such as fraudulent bidding and contracts, price-fixing, and group boycotts. Python and R are the most frequently employed programming languages, and the use of libraries (collections of already prewritten codes users can use to optimise tasks) could be found in the Revenue Service with the use of Java, even though Python is currently the most deployed language. RobOps, in turn, was programmed in C +  + and the OPS website mixes Vue and Bootstrap on its frontend, employs NET Core and Node.js to collect the data, and uses an API.NET on its backend. As the initiative depends on volunteers, this allows those collaborating to use whichever programming language they prefer.

In terms of outputs, although initiatives like Sisam and Bem-Te-Vi already deliver full written reports using natural language, a clear preference for dashboards was observed, followed by instant messages to communicate findings. For example, while *Cérebro* provides only a dashboard, Alice’s daily findings on incoherent public bids are sent daily by email to auditors and can be visualised on a dashboard, allowing auditors to take preventive action in the time window between the bid notice, the submission of the bid and the bidding award/contract signature. In the case of civil society initiatives, social media are used to make their findings visible and attract support from people, mainly using bots to invite them to help with their findings. This is the case for Rui Barbot, which identifies procedures at the Supreme Court that have been waiting to be heard for months and years, and Rosie, the AI that analyses Brazilian congresspeople’s expenses and spots suspicious spending, both of which post automated tweets. OPS uses a more conventional website with a searchable dashboard, and the YouTube and Telegram channels to publicise findings and engage users.

### Main tasks and actions

No cases of automated decision-making without human supervision were identified among the 31 initiatives analysed. The tools offer data collection, data storage, analytics, and visualisation features, along with communication features with different degrees of automation. Prediction is still underdeveloped, although the tools present risk rates. Hence, even if the initiatives use different databases and offer a different array of outputs, from dashboards to automatic email messages to chatbots and Twitter bots, they all serve to invite humans to act. In addition, they apply rules to verify and track transactions and procedures, based on what type of conduct is and is not allowed. According to the interviewees, the rules for the anti-corruption algorithms created by those developing codes are often based on existing legal norms and on common practices used to circumvent these same norms. Rosie, for example, holds the Lower Chamber accountable but not the Senate because the former has clearer formal written regulations of how representatives can spend their quota for parliamentary activity. Concerning congressional members’ expenditures, for example, the Lower Chamber does not allow representatives to pay for meals for others, and alcoholic beverages are forbidden. To Interviewee 3, clear rules like these allowed Rosie to be programmed to identify potential suspicions of illicit expenditures when the bot finds receipts listing beer and wine, and more than one full-course meal. Part of the limitation of AI-ACTs has to do with access to data and a high level of dependence on legal norms and breach of norms to classify an act as suspicious.

### Developers, users and accountability

As already noted, outsourcing anti-corruption tools is not a common feature of the initiatives under analysis. Developers are often tech-savvy civil servants, activists and citizens who want to create something useful with their tech skills. Users of these tools, in turn, can be internal or external. Apart from the two chatbots (Zello and Cida), all top-down initiatives are developed for internal use, to support civil servants’ work. Some initiatives use human action to train algorithms, such as Agata, designed for generating textual analysis with learning and developed by the TCU, which applies an active ML process based on keywords most searched for by auditors. Agata asks the auditor to say whether the result was what the person was looking for and learns by considering the context in which the word is being used. The process was constructed as a game in which the user gained ‘agatas’ (agate stones) to engage them.

Bottom-up initiatives are more likely to be designed for external users (*Tá de Pé*, Dados JusBR). The OPS’s RobOps, however, uses bots’ findings as a starting point for human accounting, conducted by the initiative’s volunteers. Interviewee 7 highlights how, in the case of the OPS, a considered decision was made not to rely mostly on machines, as the initiative considers human action as key to fighting corruption from below. The interviewees made it clear that developers perceive resistance among users, especially civil servants. Interviewee 8 said that auditors had complained that their email boxes were overloaded with Alice’s messages and the lack of staff in the TCU did not allow them to check all the alerts made by the bot. In the Federal Revenue Service, initially, inspectors had a low level of trust in the outcomes offered by Sisam. Despite the initial scepticism, those using Sisam started relying on it once they realised the number of its correct predictions had improved. Table 6 summarises the main categories and codes that emerged from the interviews regarding the human layer of AI-ACTS.

**Table 6:** Qualitative data analysis: a content analysis of in-depth interviews

Categories	Codes	Responses
Human–machine relationships	Support human work	‘The systems work together with human specialists and the observed effect comes from a human–machine combination. Humans have a good deal of freedom and are not obliged to optimise any particular measure. This makes each result more complex to present.’ (Interviewee 1)
		'What I want is to have the technology helping me to a certain extent because I want the other part to be done by society. (…) So I can't have technology that does everything for me and gives me everything ready. No. I need technology to help me. It is just like a car. The interesting thing is that you have a good vehicle, but you know how to drive it, that you can give it directions. So this is what I think of society.’ (Interviewee 7)
	Overall distrust of machines	‘Humans like to feel unique. At least here in Latin America, we like to tell our story and hear from whoever is listening something like, ‘Let’s see what I can do to help you.’ […] It is very common to see people typing or calling ‘human, human, human’ when talking to machines until a human appears to listen to their demand.’ (Interviewee 5)
Bias and unfairness	Acknowledging controversies	‘For the future –I don’t know if you’ve seen *Minority Report*, the movie, but anyway – I’m totally okay with a pre-crime unit. (…) We already do it with credit risk, when you go to the bank to get a mortgage, the bank has a model there that says whether your risk is low or high and, based on that, they decide whether you will get the loan or not. So, I don't have a lot of problems with using that [AI to fight crime]. I know there is an American state where there is some software there that tells the judge what the chances of reoffending are. (…) There is a huge controversy because “Wow, look, you're prejudging and I don’t know what, blah blah blah…”. But if it is for reducing crime rates, screw it. (…)’ (Interviewee 6)
	Lack of ethical debate	'In fact, we haven't had any ethical discussions until today, as far as I can remember. (…) Ethical concerns, in this sense, I don't think so. I don't remember a moral conflict because of some variable that I used, let's say. It is a concern that is generally missing. (…) I have seen debates like if a certain result can cause some harm to someone. We have the concern of generating serious evidence only if we really have a very high precision of the data we are receiving.' (Interviewee 8)
Auditability	Importance of open codes	‘There was a time when someone criticized us (Operation Love Serenade) because I was partisan, leftist, on Twitter (…) But it was very nice because I answered it and other people too. People said, "(He) may be a leftist, but I can see in your bio that you are in the technology area. So where is the code, where is the algorithm, the functioning logic of the program (Rosie) that puts this partisan bias you're saying it exists? The code has been open for three years. If no one has found bias so far, you could be a great pathfinder." The guy disappeared (…) The fact that the code is open brings a lot of good things. It has this side of contributions, and it has this side of auditing. Anyone can get hold of it and audit our work.’ (Interviewee 3)

As Table 6 illustrates, not only the resistance to AI-ACT among users had been observed as an issue to overcome. The lack of full transparency regarding the architecture and algorithms in the case of top-down initiatives, sometimes with the argument of security reasons, generates doubts regarding how auditable these tools are. While civil society initiatives tend to be open-source projects, governmental initiatives are treated as sensitive, although the degree of secrecy varies. Overall, this study found that classified information is less related to the databases deployed than to the algorithms, i.e., the set of rules used in calculations to highlight risks and suspicious cases. By law, even if the codes are not open, they could be audited by both internal and external control agencies. In practical terms, however, civil society is kept completely out of the process of holding these tools and their developers accountable.

Risk of bias was observed, not only because of the low level of transparency or the use of closed codes but because of the quality of data input. Iris, for example, relies on Google Street View, which tends to be outdated. Bottom-up initiatives can face issues related to bias. Rui, in turn, monitored procedures that were not randomly selected but listed by a team of journalists who selected classifiers. Unfortunately, the risk of bias is not an issue treated as a priority by the developers interviewed for this article. Interviewee 8, for example, participated in the creation of at least two AI-ACTs. When asked if he had any concerns about biases or if he thought about how a certain audit trail could offer a risk of bias, the answer was a long pause followed by ‘No’. Overall, interviewees acknowledge that academic discussions on this aspect take place, although they are not engaged with them and the discussions do not guide their work.

### Tangible outcomes

It was observed that the AI-ACTs analysed in this article focus on administrative processes by speeding up tasks, assisting decisions and supporting investigations. ContÁgil, for example, uses techniques such as clustering, outlier detection, and topic discovery functionalities that are mainly related to attempts to identify fiscal fraud and money laundering. They proved to be extremely useful in the anti-corruption probe Operation Car Wash (Lava Jato) by identifying the complex network of intermediaries and shell companies and linking them to politicians, top-level civil servants and businessmen.^8^ ContÁgil can perform in one hour what a human inspector would take a week to do and can cross multiple internal and external datasets and build network graphs indicating the level of relationship among people and companies (Jambreiro Filho, 2019).

Rosie identified over 3,000 suspicious expenditures in three months and its creators organised a sprint to formalise requests for investigation in the Lower Chamber. They reported 629 doubtful reimbursements against 216 representatives. Irregular spending on alcoholic beverages and the submission of expense receipts showing the equivalent of the consumption of 12 kg of food in a single meal were reported. Politicians went public to apologise and promised to pay the money back. The OPS Institute claim that their civic audits, conducted with the help of their bots but involving human action, resulted in saving 6.2 million reals (around 1.2 million USD). Both Rosie’s and RobOps’ accounts on Twitter, however, do not attract as many retweets and likes as their creators would like. The same happens with the Twitter bot of *Tá de Pé*. The Dados JusBR webpage has a few users, but its monitoring resulted in a report showing that the level of transparency and accessibility of prosecutors’ earnings across the states is extremely low; their work and findings gained visibility after being published in the most important Brazilian media outlets in May 2022. These initiatives, however, struggle to convince people to use their tools and to engage the public in collective actions to promote transparency, increase accountability and, hence, fight corruption.

Most developers, however, keep looking at accuracy rates to measure the efficiency of their tools, even knowing that the generated alerts may overflow email boxes or be ignored by those who are expected to act. In this regard, for example, Dataprev’s tool to detect irregularities in social benefits offers 85% accuracy. Alice, in turn, had 88.27% accuracy during the test phase and, later, during further auditing, 73.91%, but it has already helped auditors to curb irregular public hiring throughout the country. Thanks to the bot, in Goiás state, two calls for tenders for a construction project were suspended, and in Roraima state, a governmental agency was forced to redo the bid notice. Alice has verified irregular tenders at the Minister of Foreign Affairs and for restorations with resources from the Institute for National Historic and Artistic Heritage (Iphan).

Yet, a point of attention needs to be the current high level of expectation deposited in the AI-ACTs and what solutions they can offer. The creator of project FARO, Fernando Sola (2021), made a statement at a public event broadcast on YouTube and his perspective shows a certain degree of frustration: ‘When we started the work, our expectation was this: how cool, I'm going to do an AI to decide whether a complaint is suitable or not [for further examination]. We are going to have many people free to do other things. The reality is not like that. The reality is that in some cases we can do very well, give good and effective grades for these complaints, and say whether they can be investigated or not and in others we need someone to make that decision. Now, I see AI much more as an aid in most cases than as an entity making decisions practically alone’ (Fernando Sola, 2021). Based on all the findings discussed here, Sola’s testimony can be seen as a summary of the current stage of expectations and opportunities created by and the limitations of AI-ACTs, at least in Brazil. In addition, the impacts of AI applications on corruption are hard to measure because they often have indirect effects, such as automating and standardising administrative tasks that could reduce human interaction and potential corrupt exchanges. At the same time, they increase the capacity to process and find suspicious transactions.

## Conclusion

This exploratory study not only confirms that, as noted by the existing literature, Brazil is a rich source of empirical data on AI-based initiatives but also acknowledges how they remain understudied, regarding anti-corruption tools. The proposed analytical framework proved to be useful in providing an overall panorama of a wide range of techniques, functionalities and types of corruption being targeted by AI-ACTs in place in the country. One of the main positive findings is that AI-ACTs were designed to fight corruption in key governmental functions, although these tools are more likely to be tailored for specific areas.

The Brazilian cases indicate that most AI-ACTs operating in the country are related to public spending and focused on public procurement auditing, mainly due to the type of digital data available and the fact that many courts of accounts are developing these tools. Indeed, the type of corruption being combated, even indirectly by automating procedures or increasing transparency, depends on who developed and uses the AI-ACT. In the case of top-down initiatives, each tool’s focal point is linked to the formal roles of the governmental law enforcement agency deploying it. Despite a prevalence of tools to identify risks and monitor public spending, the focal point of bottom-up initiatives tends to be wider. They vary from exposing corruption procedures against politicians and delays in the Supreme Court to misuse of public money by congressional representatives to crowdsourcing information related to delays in the construction of schools.

The predominance of in-house developed tools shows how tech-savvy civil servants and the public are increasingly engaging with digital data and contributing to data-driven efforts to fight corruption. Improvements in the quality and easier access to technology and the large volume and variability of digital data, mainly open and public data, can be seen as the drivers behind the current “AI spring” in anti-corruption in Brazil. Most AI-ACTs have been designed to support human action in examining suspicious cases rather than creatively spotting new types of corruption and solving problems on their own. This feature often brings out issues such as understaffed agencies and overburdened citizens becoming overloaded with information and pays less attention to suspicious findings spotted by the bots. This proved to be the case in the Federal Court of Accounts, as previous studies had also noted. And the bottom-up initiatives analysed here experience decreased engagement over time.

Even if the bots we have now are more likely to be efficient in showing where humans should be paying attention than in decision-making, there is a low level of accountability of the algorithms and, hence, a high risk of bias. Due to the lack of transparency of the top-down initiatives, there is no information on whether the automated systems are replicating already existing bias and discriminatory patterns. Bottom-up initiatives, in turn, tend to use open codes but have a high dependence on open data, which is not always available or accessible, compromising their accuracy and scope. Past cases and existing legal norms are the basis for classifying certain acts as suspicious, which means that algorithms may be reproducing a prevailing bias. This limits the scope of initiatives and makes it more difficult to capture non-illicit acts, i.e., those that do not deviate from the formal norms but capitalise on abuses of collective trust and result in injustices and particularism.

Little information is available on AI-ACT outcomes regarding measurable impacts on corruption, although the findings suggest that some resulted in positive results, such as misused public money being paid back and supported audits and criminal investigations. More research on both the impacts and the new measurements for calculating outcomes is needed. How AI-ACTs should be held accountable, who controls and oversees them, and what types of structures of power and bias they are subjected to are questions that are also worth addressing. What triggers the creation of such initiatives, how AI has been used and rejected, and whether they are working to serve its main purposes in the fight against corruption are crucial topics for future research. If we want better anti-corruption efforts, we need to understand how these tools operate, their impacts and risks, who created them, and who is monitoring the monitors. Otherwise, we risk perpetuating the ills we seek to cure. Until now, AI-ACTs are being developed faster than we can critically comprehend and, most importantly, assess whether they are fair, meaningful and technically feasible. This is certainly the case in Brazil, but it is likely not the only place.

## Data Availability

The datasets supporting findings are included in this published article and its supplementary information file. Part of the data not publicly available due to the fact that they constitute an excerpt of research in progress could be made available upon reasonable request and with permission of the ERC funded BIT-ACT (Bottom Up Initiatives and Anti-Corruption Technologies) project.

## Appendix 1

Table

**Table 7:** List of AI-ACTs in use in Brazil mapped for this study (April 2021)

AI-ACT	Launch	Layers		
*Name*	*Year, by*	*Technological infrastructure*	*Functional*	*Human (auditability and bias)*
*Projeto Cérebro (Brain Project)*	2015, Council for Economic Defense (CADE)	Using public databases related to ownership register and public procurement, it applies data mining, descriptive statistics, and economic filtering with R and Python to generate a dashboard signalling any potential risk and/or sign of violation of competition law	Improving horizontal accountability mechanisms and supporting human action in the Council of Economic Defense with analytics and visualization. No machine learning technics are in place yet	Auditable (internally and by control agencies), but not open to the public. No info about bias, although there is a risk due to the data and processes used
*Rosie and Jarbas*	2016, Operação Serenata de Amor (Love Serenade Operation)-Open Knowledge Brasil	A Python-programmed application that first applies hypothesis and test-driven development processes and then unsupervised learning algorithms to estimate a “probability of corruption” based on standard deviations for each reimbursement receipt submitted by MPs. Rosie processes public and private open databases made available by the Lower Chamber, Revenue Services, Google, Foursquare, and Yelp. Findings are on Rosie's Twitter account and on a dashboard named Jarbas	Improving social accountability by inviting people to check suspicious cases, after auditing MP expenditures with machine learning techniques and communicating findings via Twitter and online dashboard with filters	Auditable, open-source GitHub hosted. Not free from bias as data are clustered and standard deviations are used in the analysis
*Rui Barbot*	2018, JOTA news website	A crawler created in Python to collect public and open data of hundreds of procedures on the Supreme Court website. It checks when each one was updated and, if any procedure is reaching 180 or 270 days or years without movement, Rui tweets automatically about the anniversaries and informs its findings by emailing journalists from JOTA, the news outlet that created the bot	Improving social accountability by calling attention to bottlenecks in the Brazilian Supreme Court by using analytics and communication tools to signal which procedures are facing sluggish progress	Auditable. Risk of bias as the list of monitored procedures and classifiers to measure delay were not randomly selected, although they are based on journalistic criteria
*Esmeralda*	2019, Court of Accounts of the Municipalities of the Goiás State (TCM-GO)	Esmeralda processes data received through a system created to transfer data from and to the municipalities in the State of Goiás audited by the local Court of Accounts. Using both open and protected data, it identifies anomalies related to the purchase and payment of goods, services, workers, and benefits using data mining and statistical analyses. Auditors can access the data and analytics on their desktops. Findings are also transferred via Elasticsearch, a free and open search and analytics engine. Notifications of risky cases can be sent to auditors via internal chat	Improving horizontal accountability mechanisms and supporting auditors' action with visual analytics and communication tools	Auditable internally and by control agencies, but not open to the public. No info about bias, although there is a risk due to the data and processes used
*Iris (Indicator de Risco de Irregularidades em Contratações, or Irregularities Risk Indicator of Public Contracts)*	2015, Court of Accounts of the State of Rio de Janeiro (TCE-RJ)	Iris applies a multicriteria model based on an analytical hierarchy process to establish relationships and calculate risk factors by using data mining, deep learning, and image processing. As inputs it uses sensitive, protected, and open data provided by municipalities and the State of Rio de Janeiro related to public procurement, along with data on business ownership and aerial and street-level images. Among the processes in place, Iris uses Google services to convert addresses into geographic codes and to generate aerial and street-level images of the companies that are participating in the bidding. Based on these images, a model applying a convolutional neural network (CNN, or ConvNet) evaluates the probabilities of each participant being a ghost company. Ready-to-use libraries and image recognition models (e.g., TensorFlow and Inception v3) are used. As outputs, Iris offers a spreadsheet indicating the percentage of risk of each of nine risk factors linked to detailed reports that can be accessed through a web browser	Improving horizontal accountability mechanisms and supporting auditors' action with visual analytics and communication tools	Auditable (internally and by control agencies), but not only it is closed to the public, but TCE-RJ is not very transparent about Iris. Even overall information on inputs and outputs and software used are classified as sensitive and were not made available via Access to Information Law. There is a high risk of bias and inaccuracy, e.g., Google Street View tend to be outdated
*DIB (Detecção de Irregularidades em Benefícios, or Detecting Irregularities in Benefits)*	2018, governmental Social Security Technology and Information Company (DataPrev)	Multiple-technique tool for benefit fraud detection that uses as inputs governmental datasets (with protected social, labour, and pension governmental data) and deploys data mining and statical analyses with Python and SAS Miner and rules engine with audit trails already known or defined in the data exploration stage. It also uses techniques such as deep learning by applying CNN. The algorithm was trained based on a sample of 3,000 to 6,000 benefits, with 85% accuracy. It offers panels and storyboards to identify patterns and signal risks of fraud and suspicious cases, using data visualization applications, such as React and SpringBoot, georeferencing libraries (leaflet) and QlikView	Improving horizontal accountability mechanisms and supporting inspectors’ action with visual analytics and communication tools	Auditable, but not open to the public. Internal and external control units could audit it. The risk of bias depends on the quality of data available and the models adopted
*Alice (Análise de Licitações e Editais, Analysis of Biddings and Call for Bids)*	2015, Office of the Comptroller General (CGU) and later improved by the Federal Court of Accounts (TCU)	Programmed in Python, Alice reads tenders published on Comprasnet (public procurement portal) and the Federal Official Gazette daily looking for keywords and values that will allow it to identify signs of irregularities based on previously programmed audit trails. It mixes text pre-processing, data mining, machine learning (Random Forest, Support Vector Machine, Bernoulli Naive Bayes) and regular expressions techniques. Alice also crosses data, including names of suppliers, products and prices aiming to identify, for example, competing companies that have common owners or previous issues related to public procurement. It applies supervised learning to create a classification of risk, allowing auditors to act preventively in the time frame between the bid notice and the submission of the bid and before the bidding award/contract signature. It offers communication and data visualization tools with automatic daily emails with alerts and a dashboard with overall and detailed information on each suspect case	Improving horizontal accountability mechanisms and supporting inspectors’ action with alerts, visual analytics and communication tools	Auditable, but not open to the public. Internal and external control units could audit it. The risk of bias depends on the quality of the data available and the models adopted. Accuracy was 88.27% during the test phase and, later, during further auditing, it was 73.91%
*Agata (Aplicação Geradora de Análise Textual com Aprendizado, or App for Generating Textual Analysis with Learning)*	2020, Federal Court of Accounts (TCU)	Using data available on Comprasnet (governmental procurement portal), it is an active machine learning process based on keywords searched by auditors. Programmed with Python using the library that Apache Solr includes, it applies text classification and segmentation, sectorisation (converting words into numbers) and hierarchical clustering of similar texts. The auditor is requested to say if the search is what she is looking for, which trains the algorithm via logistic regression. The process was constructed as a game in which the user gains agates to engage them. When the user gets three stones, she can subscribe to receive notifications via email. It also offers a dashboard with overall and detailed information on keywords that may be of interest and more likely to require an inspection	Improving horizontal accountability mechanisms, engaging with and supporting inspectors’ action with alerts, visual analytics and communication tools	Auditable, but not open to the public. Internal and external control units could audit it. The risk of bias depends on the most frequently searched words and their different meanings
*Monica (Monitoramento Integrado para o Controle de Aquisições. Integrated Monitoring for Procurement Control)*	2017, Federal Court of Accounts	It deploys data mining and statical analyses to look for anomalies in public procurement in the federal legislature, judicial and executive branches and the prosecution service. It offers data visualization tools with filters and the possibility to download spreadsheets	Improving horizontal accountability mechanisms and supporting inspectors’ action with alerts, visual analytics and communication tools	Auditable, but not open to the public. Internal and external control units could audit it. There is a risk of bias
*Adele (Análise de Disputa em Licitações Eletrônica, Dispute Analysis in Electronic Bidding)*	2018, Federal Court of Accounts	As inputs, it uses protected and sensitive data on electronic biddings, such as IP addresses of the bid participants and data on companies and individuals. Data mining allows informing inconsistencies and anomalies that are informed in the form of a dynamic dashboard	Improving horizontal accountability mechanisms and supporting inspectors’ action with alerts, visual analytics and communication tools	Auditable, but not open to the public. Internal and external control units could audit it. There is a risk of bias
*Sofia (Guidance System on Facts and Evidence for the Auditor)*	2020, Federal Court of Accounts	Available to the TCU auditors, Sofia works as an extension that reviews draft texts by verifying sources of reference and identifying the correlation between the information written in the text and other procedures, e.g., names, IDs and fiscal codes. It informs, for example, if a person is dead or under investigation or has been convicted in the Court of Accounts. The information appears in comment boxes along the text	Improving horizontal accountability mechanisms and supporting inspectors’ action with alerts, visual analytics and communication tools	Auditable, but not open to the public. Internal and external control units could audit it. There is a risk of bias. If biases are not considered, the tool may generate misinterpretations and negative impacts on auditing
*Carina (Crawler e Analisador de Registros da Imprensa Nacional)*	2020, Federal Court of Accounts (TCU)	It uses data crawling, along with machine learning and regular expressions techniques applied to analyse data daily from the Federal Official Gazette as an attempt to identify anomalies related to public contracts and bids. Carina offers a dashboard and emails with information about suspicious and anomalous purchases of urgently vital health products and services needed in the response to COVID-19	Improving horizontal accountability mechanisms and supporting inspectors’ action with alerts, visual analytics and communication tools	Auditable, but not open to the public. Internal and external control units could audit it. If biases are not considered, the tool may generate misinterpretations and negative impacts on auditing
*Sisam (Sistema de Seleção Aduaneira por Aprendizado de Máquina, customs selection system through machine learning)*	2014, Federal Revenue Service (Receita Federal)	Sisam learns both from inspected and non-inspected import declarations and calculates the probability of about 30 types of errors for each declaration, e.g., false description of goods, missing licenses, wrong preferential tax claims. It is a set of Bayesian networks whose conditional probability tables have been replaced by smoothing hierarchies. The system is implemented in Java. Its inputs are sensitive and highly protected economic, financial, tax and customs datasets. As outputs, Sisam offers an interactive spreadsheet with colourful highlights. Sisam also produces a written report based on natural language explanations presenting error probabilities, alternative values for each field of import forms that can be wrong, and the return expectation of each possible inspection	Improve customs control by generating aggregated data about importers, hence helping post-clearance revisions. It supports inspectors with alerts, visual analytics and communication tools to fight fraud and tax evasion in import operations. These can be “copied and pasted” to inspection reports	Auditable, but not open to the public. Internal and external control units could audit it. There is a risk of bias and errors. Information on accuracy is not available. Depending on how the algorithm is trained, it may not detect some cases of fraud whereas it may raise false positives in other cases, changing the focus of the clearance procedure
*Aniita (Analisador Inteligente e Integrado de Transações Aduaneiras, Intelligent and Integrated Analyser of Customs Transactions) and BatDoc (Batimento Automatizado de Documentos na Importação, Document Mismatch Detector)*	2012, Federal Revenue Service (Receita Federal)	It uses sensitive and protected data such as import declarations and digital images of auxiliary documents, e.g., invoices and bills of lading, to identify anomalies related to possible cases of tax evasion and tax avoidance in customs. Part of the Sisam, BatDoc is a tab of Aniita. It applies optical character recognition to the digital images of auxiliary documents, identifies relevant fields and performs transformations to the data to handle differences in how these fields are presented in each document. Implemented in Java, it uses Abby, a commercial optical character recognition tool. It offers a dashboard with detected divergences in company names, addresses, prices, quantities, HS codes, and incoterm codes comparing the description of the goods in the invoices and their description in the import declaration	Support human decisions by identifying suspicious elements in imports	Auditable, but not open to the public. Internal and external control units could audit it. There is a risk of bias and errors. Information on accuracy is not available. Depending on how the algorithm is trained, it may not detect some cases of fraud whereas it may raise false positives in other cases, changing the focus of inspectors' action
*Malha Fina de Convênios (Singling out agreements for inspections)*	2018, Office of the Comptroller General (CGU)	A predictive model created by the CGU indicates with a degree of certainty whether accounts presented by those who received federal funds can be rejected or not accepted at the moment they are presented for examination and settlement. Written in Python, it deploys automatized analysis of 104 variables using supervised machine learning (Random Forest) and uses public and protective data available on Siconvr and Plataforma + Brasil platforms, along with the alerts of possible irregularities signalled by other tools developed by the CGU. It provides a score to measure the likelihood of having accounts rejected or not (1 to 0)	Supporting human decisions by identifying elements of misuse of federal funds	Auditable, but not open to the public. Internal and external control units could audit it. There is a risk of bias and errors. Information on accuracy is not available. If biases are not considered, the tool may generate misinterpretations and negative impacts on rights
*Faro (Ferramenta de Análise de Risco em Ouvidoria, or Risk Analysis Tool for Ombudsman)*	2019, Office of the Comptroller General	Faro classifies complaints and denunciations received via the Fala.BR online system (governmental crowdsourcing platform) as suitable or not. Complaints considered suitable meet the minimum requirements to justify the opening of an investigative procedure. Programmed in Python, it uses supervised machine learning techniques (ensembles of decision trees) and textual analyses and crosses information with data already available in investigative procedures and other database storage at the CGU. Around 3,500 registries were used to train the algorithm. As outputs, it offers suitability for investigation (score from 0 to 1)	Supporting human decision. Complaints with high scores, i.e., considered suitable, are sent to inspectors to open investigative procedures. The ones Faro considers not suitable are sent back to the complainant, asking for supplementary information	Auditable. The model is auditable by the development team and outcomes by the business area (ombudsman). Cases with a high degree of doubt are analysed by auditors. Depending on how the algorithm is trained, it may not detect some cases of fraud whereas it may raise false positives in other cases, changing the focus of the investigation
*Watson*	2017, IBM	Watson works as a text indexing tool of all databases available at the federal police in the State of Rio Grande do Sul and allows searches regarding investigative procedures based on data mining, natural language processing and classification techniques. It was written in Java and XML and uses non-supervised learning. It offers an output dashboard with questions and answers	Facilitate the work of police officers	Auditable by the federal police in the State of Rio Grande do Sul. There is a risk of bias. When not analysed for possible bias detection, may impact negatively on the presumption of innocence principle
*Delphos*	2020, Federal Police (PF)	Using natural language processing, Delphos makes predictions and signals risks, including the risk of fraud, by recognizing and crossing names of people, companies, addresses, values of contracts and payments, emails, and telephone numbers. It uses public and protected databases	Facilitate the work of police officers	Auditable, but not open to the public. Internal and external control units could audit it. Natural language processing tools used to predict risks related to criminal actions may impact negatively on marginalized populations. The algorithm may easily be biased against low-income people who do not know Portuguese well or those who use informal language, abbreviations and certain types of slang
*Cida (Chatbot Interativo de Atendimento Cidadão, or Interactive Chatbot for Citizen Service)*	2018, Office of the Comptroller General (CGU)	Although the CGU Cida does not use AI techniques, it is a chatbot implemented in Java that interacts with Fala.BR platform through the web and uses the Facebook and Telegram libraries to communicate with users. It guides users who want to enter complaints, denunciations, or greetings or give feedback	Offering opportunities for citizen engagement on social media and opening a dialogue channel. Cida automatically registers the communication on the Fala.BR platform	Auditable, but codes or interactions are not open to the public. Internal and external control units could audit it. It cannot work well with low-income people who do not know Portuguese well or those who use informal language
*Zello (chatbot)*	2018, Federal Court of Accounts (TCU)	Zello conducts online chat conversations via text and uses supervised learning and NLP e Named Entity Recognition techniques. It offers options such as access to procedures based on names provided by users and it also issues clearance certificates. Developed by the TCU, Zello initially used direct messages on Twitter and is now on WhatsApp, interacting with citizens via text messages. It was launched in 2018 to facilitate access to the list of mayors and governors with public spending accounts declared irregular. Since then it has been adding functionalities, such as consulting procedures and actions related to COVID-19 responses	Offering opportunities for citizen engagement on social media and opening a dialogue channel	Auditable, but codes or interactions are not open to the public. Internal and external control units could audit it
*Dados Jus Br*	2020, Federal University of Campina Grande and Transparência Brasil	Organizes and unifies data on salary and top-up salary payments in the bodies that make up the Brazilian justice system. Using R, Java, Python, Go and Vu, it automatized the collection of data and offers them in a searchable dashboard with graphs and tables	Improving social accountability and offering organized access to information often available in different formats and platforms	Auditable, open-source GitHub hosted
*Ta de Pé Merenda and Compras Emergenciais*	2020, Transparência Brasil and Federal University of Campina Grande	Extraction, processing, and storage of data from two State Courts of Accounts (Rio Grande do Sul and Pernambuco), and the Federal Revenue Service using TypeScript, Java, Python, R and HTML. It highlights contracts and services with irregularities in a dashboard	Improving social accountability by offering detailed information on contracts with issues detected at the municipal level	Auditable, open-source GitHub hosted
*﻿CoviData*	2020, Federal Court of Accounts (TCU)	Makes predictions and provides risk scores based on public and sensitive databases and pieces of news. It deploys a language representation model called BERT, which stands for Bidirectional Encoder Representations from Transformers, for supervised learning to identify risks	Improving horizontal accountability and supporting human decisions by identifying suspicious elements and scoring the risks	Auditable, but not open to the public. Internal and external control units could audit it. There is a risk of bias and errors. Information on accuracy is not available
*PLACC (Plataforma de Análises Cognitivas para o Controle, or Cognitive Analytics Platform for Control)*	Year not available, Federal Court of Accounts (TCU)	Models that use as input texts of court documents and semi-structured metadata are available on the federal justice website, by applying the conditional random field (CRF) and long short-term memory (LSTM) techniques. The main goal is to identify individuals and companies, extract relevant information about them and establish relationship networks and money flows	Improving horizontal accountability and supporting human decisions by identifying suspicious relationships and connections	Auditable, but not open to the public. Internal and external control units could audit it. There is a risk of bias and errors. Information on accuracy is not available
*Ta de Pé*	2017, Transparência Brasil	A mobile phone application that was later converted into a WhatsApp chatbot, it was developed for citizens to monitor school construction projects in Brazilian municipalities. The user submits data about construction sites that are behind schedule or with no work in progress. Such crowdsourced information is sent to independent engineers, and Transparência Brasil contacts the mayors’ offices about project delays. A Twitter bot posts a message each time a user submits a new picture for evaluation, or a municipality responds to a citizen’s request	Fostering bottom-up accountability in the Brazilian public sector and improving responsiveness in government education expenditure	Auditable, but codes and interactions are not available on the Transparência Brasil website. An academic study showed that the app has a null impact on school construction indicators and that politicians are unresponsive to individual requests
*Ajna (Plataforma de Visão Computacional e Aprendizado de Máquina, Computer Vision and Machine Learning Platform)*	2017, Revenue Service	Implemented in Python, it uses the TensorFlow library for deep neural network and SciKit-Learn and Random Forest regressors to estimate potential risks and threats regarding the goods observed in x-ray images in the port of Santos. All containers leaving or entering the country are scanned. Ajna collects the resulting images, associates them with the corresponding declarations and makes the images available to customs officers in their web browsers whenever convenient. Hence, Ajna applies computer vision, data mining and optical character recognition techniques for classifying, predicting and scanning patterns and warning of anomalies aiming, among other things, to control fraud and improve functional behaviour and workers' security in customs clearance procedures	Improving horizontal accountability and supporting human decisions by identifying suspicious elements in imports and exports	Auditable, but not open to the public. Internal and external control units could audit it. There is a risk of bias and errors. Information on accuracy is not available
*ContÁgil*	2009, Revenue Service	It uses decision trees, naive Bayes, support vector machines and deep neural networks and includes clustering, outlier detection, topic discovery and co-reference resolution features for retrieving and analysing sensitive and protected data. It is available to all Revenue Service employees through an interactive graphic interface. All ContÁgil functions are also available through scripts that can be created visually or written in Javascript or Python. It reads account books and invoice sets, scans the various data sources to which it has access and builds network graphs with people, companies and their relationships	Supporting human decisions and improving identification of big fraud schemes like the one observed in Operation Car Wash	Auditable, but not open to the public. Internal and external control units could audit it. There is a risk of bias and errors Information on accuracy and data inputs is not available
Publique-se	2020, Abraji, Transparency International Brazil	Programmed in Python, it automatically checks if there are legal proceedings that cite politicians as defendants or plaintiffs in the higher, federal and local courts. It has already identified 3445 politicians. It has the option to download the list of procedures related to active and passive corruption and administrative improbity. It was developed by a company called Digesto, which specialises in law technology	Supporting human action, mainly journalistic work, to check criminal and civil procedures linked to politicians	Auditable, open-source GitHub hosted
Bem-Te-Vi	2020, TST	Information from the labour legal language model, built from data coming from the 27 Regional Labour Courts, justice decisions in the past two years. ﻿Deploys Word2vec, AutoML, and XGBoost to predict decisions and proceedings and select the most experienced assistant to analyse court cases. It took two years to develop	Supporting human action to speed up procedures and reduce eventual conflict of interests	Auditable, but not open to the public. Internal and external control units could audit it. There is a risk of bias and errors
RobOps	2016, Instituto OPS	Programmed in C#, Vue and Java Script, RobOps works daily, collecting data on the Lower Chamber and the Senate, crosschecks with fiscal conditions at the Revenue Service and displays the expenditures raking the politicians in a dashboard. Its finds are also communicated by email. The process allows volunteers to analyse in-depth the spending. New functions have been added to improve both dashboard and automated crawling	Fostering bottom-up accountability in the Brazilian congress and improving responsiveness to public spending by the legislature	Auditable, open-source GitHub hosted

Sources: Author based on Access of Information Answers; Interviews; GitHub (https://github.com/analytics-ufcg/ta-de-pe-dados; https://github.com/dadosjusbr; https://github.com/okfn-brasil/serenata-de-amor; https://github.com/ops-org; https://github.com/RafaelEstevamReis/Robops); Costa and Bastos (2020); Agata 2020 (https://www.youtube.com/watch?v=1extYx6VWa8); 3º Seminário sobre Análise de Dados na Administração Pública 2017 YouTube (https://www.youtube.com/watch?v=Pw-DW5ptvbQ&t=5963s); Transparência Brasil/Catálago IA (2021) https://catalogoia.omeka.net/; Casos de Aplicação de Inteligência Artificial na Administração Pública—Enastic AGU 2020 https://www.youtube.com/watch?v=2chdQSjM2GI; Freire, Gaudino, Mignozzetti (2020); Transparência Brasil (2021a, 2021b)—https://www.transparencia.org.br/downloads/publicacoes/Governance_Recommendations.pdf; Jambreiro Filho (2019), Coutinho (2012)

7

## Footnotes

1 *Tá de Pé* is an informal Brazilian expression for ‘Is it done?’ Literally, it means ‘standing on its feet’ in Portuguese (Freire, Galdino and Mignozzetti, 2020).

2 The digital transformation of public procurement, court proceedings, and public records, along with governmental open data and transparency policies and additional new data sources generated by satellites, electronic devices, and social media, have proved to be essential in developing many AI-ACTs currently in place. However, this type of data is not always accessible everywhere with the same quality.

3 This study acknowledges, however, that improving such types of control can indirectly impact possible attempts to bribe inspectors who control mine licensing and any illegal trade.

4 MARA was developed by the Brazilian Office of the Comptroller General to estimate the risks of professional misconduct, including corrupt behaviour, among its civil servants by using dozens of variables, including how the person was employed (formal exam or political appointment), political affiliation and criminal background. It trains the algorithm based on a dataset containing details of thousands of workers punished in the past for administrative misconduct (Aarvik 2019; Köbis, Starke, and Rahwan 2022).

5 See https://catalogoia.omeka.net/. Accessed: 22 April 2021.

6 See *Achados and Perdidos* from Transparência Brasi. l (http://www.achadosepedidos.org.br/usuarios/tburg) and the governmental website *Busca de Pedidos e Respostas* (http://www.consultaesic.cgu.gov.br/busca/SitePages/principal.aspx).

7 ‘Delphos’ *Catálogo de Uso de Inteligência Artificial por Órgãos Governamentais*. Accessed on April 22, 2021, https://catalogoia.omeka.net/items/show/34.

8 Operation Car Wash was an investigation into money laundering that became public in March 2014 and quickly turned into a much greater corruption probe, uncovering a wide and intricate web of political and corporate racketeering with unprecedented repercussions not only in Brazil but in several other Latin American and African countries (Lagunes et al., 2021).
